# Health-related quality of life in breast cancer patients in low-and-middle-income countries in Asia: a systematic review

**DOI:** 10.3389/fgwh.2023.1180383

**Published:** 2023-06-14

**Authors:** Nhi T. N. Ngo, Ha Thi Nguyen, Phuong Thi Lan Nguyen, Truc Thuy Thanh Vo, Toi Lam Phung, Anh Gia Pham, Thanh Van Vo, Mai Thi Ngoc Dang, Tien Nguyen Le Bao, Khanh N. C. Duong

**Affiliations:** ^1^ School of Medicine, Vietnam National University, Ho Chi Minh, Vietnam; ^2^ Ministry of Health, Health Strategy and Policy Institute, Ha Noi, Vietnam; ^3^ Oncology Department, Viet Duc Hospital, Hanoi, Vietnam; ^4^ Department of Surgery, Hanoi Medical University, Hanoi, Vietnam; ^5^ Institute of Orthopedics and Trauma Surgery, Viet Duc Hospital, Hanoi, Vietnam; ^6^ Center of Clinical Pharmacology, Hanoi Medical University, Hanoi, Vietnam; ^7^ Department of Pharmacotherapy, College of Pharmacy, University of Utah, Salt Lake City, UT, United States

**Keywords:** breast cancer, HRQoL, QoL, Asia, LMICs, systematic review

## Abstract

**Introduction:**

Breast cancer remains one of the major cancers worldwide. In Asia, breast cancer is leading both incidence and mortality rates. Health-related quality of life (HRQoL) studies play an important role in clinical treatment. This systematic review aimed to summarize the evidence of HRQoL and associated factors among patients with breast cancer in low-and-middle-income countries (LMICs) in Asia.

**Method:**

Performed according to PRISMA guidelines for systematic review, the studies were searched from three databases (PubMed, Cochrane, Scopus) up to November 2020. The studies which met the predefined eligibility criteria were selected, extracted, and assessed the quality according to the Newcastle—Ottawa Scale (NOS) tool.

**Results and Discussion:**

A total of 2,620 studies were searched on the three databases, of which 28 met the selection criteria, then, were included in the systematic review. The Global Health Status (GHS) score of breast cancer patients based on the EORTC QLQ-C30 questionnaire ranged from 56.32 ± 25.42 to 72.48 ± 15.68. The overall HRQoL scores using the FACT-G and FACT-B instruments ranged from 60.78 ± 13.27 to 82.23 ± 12.55 and from 70.29 ± 13.33 to 108.48 ± 19.82, respectively. Factors affecting HRQoL of patients with breast cancer included age, education level, income, marital status, lifestyle, tumor stage, method, and treatment duration. Patient's income showed a consistent effect on HRQoL while the remaining factors reported inconsistent findings across the studies. In conclusion, the HRQoL of breast cancer patients in LMICs in Asia was low and affected by several sociodemographic factors which should be studied more in future research.

## 1. Introduction

Female breast cancer is the most common cancer globally (1). According to GLOBOCAN 2020, there were 2.3 million new breast cancer cases and 685,000 deaths worldwide (2). In Asia, breast cancer is the leading cancer in both incidence and mortality rates, with 1.03 million new cases and 346,000 deaths, accounting for 45.4% and 50.5% of the global figure, respectively (2). Globally, although the incidence rate was 88% higher in developed countries, the mortality rate was 17% higher in developing countries (3). This trend was also found in the East Asia and Pacific, Europe and Central Asia, and South Asia regions, where high-income countries (such as Singapore, Japan, South Korea, Brunei, and Israel) had significantly higher incidence rates, but lower mortality rates compared to low-and-middle-income countries (LMICs) (4). The burden of breast cancer has been increasing in both high-income countries (5) and LMICs in Asia (4–6); however, LMICs face significant challenges as they account for 70% of deaths due to breast cancer (7). This is largely because the majority of breast cancer patients in these countries are diagnosed at advanced stages (4). Breast cancer also poses an onerous financial burden on patients and their families in these countries. A study in India showed that 30% of breast cancer patients had moderate financial difficulties and had to sell or mortgage assets to continue treatment (8). In addition, 13% of the patients had severe financial problems, 10% of which had to find high-interest loans and, 3% had to discontinue treatment due to financial inability (8).

World Health Organization (WHO) defined quality of life (QoL) as an individual’s perception of their position in life in the context of the culture and value systems in which they live and also related to their goals, expectations, standards, and concerns (9). Health-related quality of life (HRQoL) is a subgroup of QoL, which solely evaluates the health-related aspects of QoL of an individual (10). HRQL is a multidomain concept that represents the patient’s general perception of the effect of illness and treatment on physical, psychological, and social aspects of life (11). Assessment of HRQoL in breast cancer patients can support physicians, healthcare providers, and policymakers in making decisions to improve patients’ outcomes (12, 13).

Ho and his colleagues conducted a systematic review of HRQoL in breast cancer patients in Asia in 2017 (14). There were additional studies about HRQoL in breast cancer patients in LMICs in Asia published after 2017 (15–20). An updated systematic review of HRQoL in breast cancer patients along with associated factors among LMICs in Asia is needed to give a better understanding of HRQoL among these patients. Therefore, this systematic review aimed to systematically synthesize the evidence on HRQoL and factors that are associated with HRQoL in breast cancer among LMICs in Asia.

## 2. Method

### 2.1. Search strategy

The systematic review was conducted according to the PRISMA Statement (21). Studies were searched on three databases PubMed, Cochrane Library, and Scopus from the database inception until November 2020. Search strategies were developed based on the following search terms, “breast cancer”, “breast neoplasm”, “breast carcinoma”, “breast tumor”, “quality of life”, “patient-report outcome”, and “HRQoL”. The details of search strategies in three databases were described in Supplementary Material S1. The reference lists of selected studies were also screened to identify further relevant studies.

### 2.2. Study selection

Two reviewers (N.N. and T.V.) independently screened titles and abstracts and examined the full texts of potentially eligible articles. Studies were included if they meet the inclusion and exclusion criteria. Inclusion criteria includes: (1) the studies were conducted in breast cancer patients; (2) the outcome was QoL or HRQoL; (3) the study setting was LMICs in Asia. Exclusion criteria consists of: systematic review, meta-analysis, pilot studies, case studies, editorials, comments, and conference abstracts. Discrepancies were resolved by a third reviewer (K.D).

### 2.3. Data extraction

Two reviewers (N.N. and P.N.) independently extracted data from all eligible studies based on the pre-designed form. The following information was extracted: author, year of publication, study characteristics (e.g., country, economic level, study design, sample size, respondent rate, participant, questionnaire); study outcome (e.g., HRQoL score, factors associated). The setting of studies was determined through World Bank’s statistics in July 2020. The classification of the countries was defined according to the Gross National Income (GNI) as below (22):

• Low-income country: the GNI is $1,045 or less.
• Lower-middle-income country: the GNI is from $1,046 to $4,095.
• Upper-middle-income country: the GNI is from $4,096 to $1,695.

The time frame for this classification was valid from 2020 and earlier.

Any disagreement was solved by discussion and if necessary, the opinion of a third reviewer (K.D) was sought.

### 2.4. Risk of bias assessment

The Newcastle-Ottawa Scale (NOS) was used to assess the quality of case-control and cohort studies (23) and the adapted version of NOS was used for cross-sectional studies (24). Two same reviewers (N.N and P.N) assessed the quality of studies independently and any disagreement was solved by discussion.

## 3. Results

### 3.1. Selection of studies

**Figure 1:**
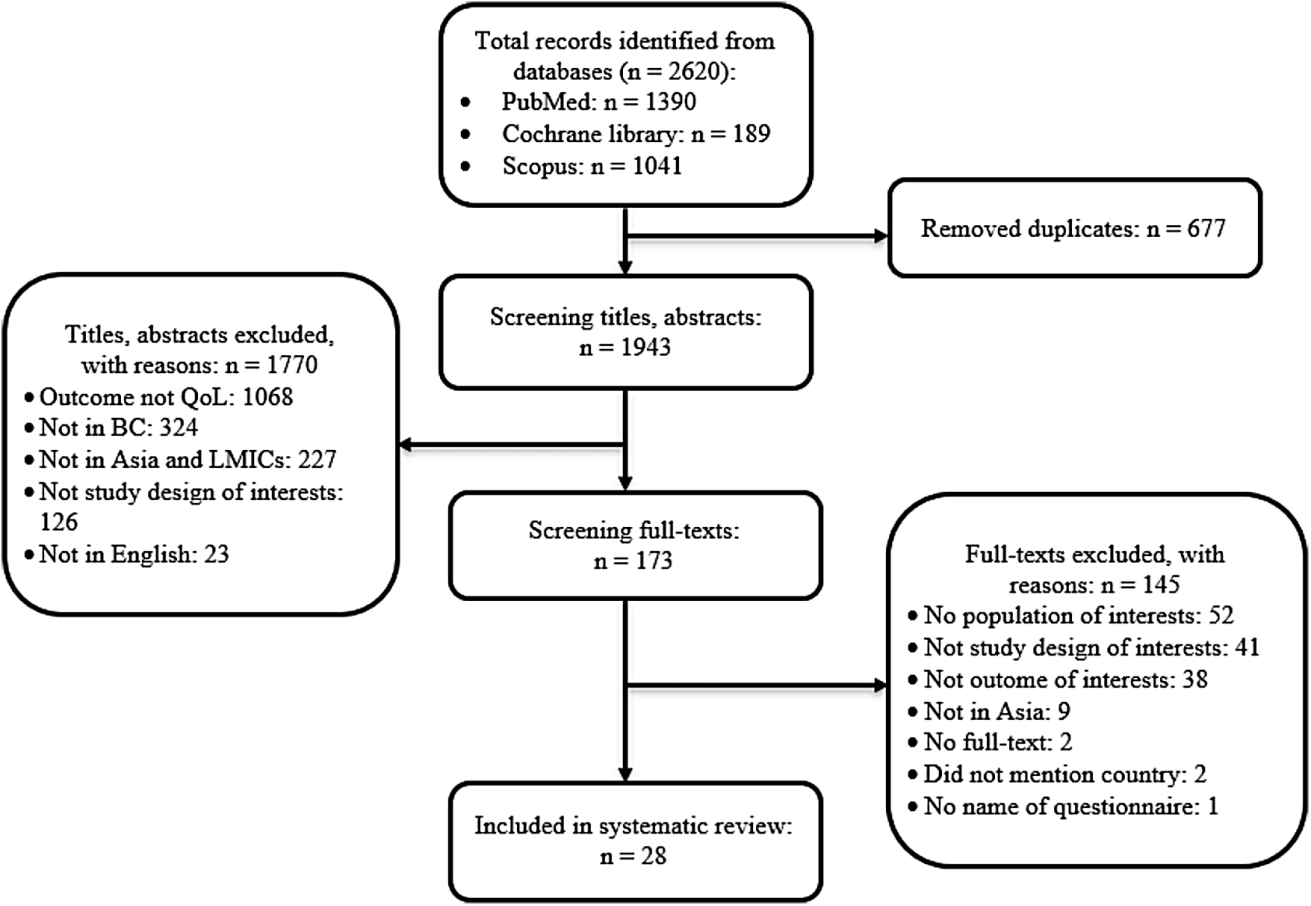
The PRISMA flowchart of selection eligible studies.

A total of 2,620 records were searched from three databases. 677 studies were removed as duplicated studies, and 1943 studies were screened with titles and abstracts. 173 out of 1943 studies were selected for full-text screening. Eventually, 28 studies met eligible criteria and were included in this systematic review. Figure 1 shows the PRISMA flowchart of study selection.

### 3.2. Characteristics of eligible studies

Table 1 illustrated the characteristics of eligible studies. Of the 28 selected studies, more than half of them were conducted in China (*n* = 15; 53.6%), followed by Malaysia (*n* = 6; 21.4%), India (*n* = 3; 10.7%), Vietnam (*n* = 1; 3.6%), Sri Lanka (*n* = 1; 3.6%), Thailand (*n* = 1; 3.6%), and Indonesia (*n* = 1; 3.6%). 23 studies were conducted in upper-middle-income countries (China, Thailand, and Indonesia) while the remaining 5 studies were in lower-middle-income countries (Vietnam, India, and Sri Lanka). 24 studies recruited breast cancer patients from all stages of disease and another 4 studies investigated newly diagnosed breast cancer patients. The sample size of included studies ranged from 70 to 10,794 with the respondent rate was from 49% to 100%. A variety of questionnaires to assess HRQoL were used across 28 studies. The Functional Assessment of Cancer Therapy—Breast (FACT-B) was the most common one with nine studies (32.1%), followed by the European Organization for the Research and Treatment of Cancer Quality of Life Questionnaire (EORTC QLQ-C30) in eight studies (28.6%), the European Organization for Research and Treatment of Cancer Breast Cancer-Specific Quality of Life Questionnaire (EORTC QLQ-BR23) in six studies (21.4%), the Functional Assessment of Cancer Therapy—General (FACT-G) in four studies (14.3%). Other HRQoL questionnaires were used with lower frequencies such as the EuroHRQoL five-dimension scale (EQ-5D), the EuroHRQoL-visual analogue scale (EQ-VAS) in three studies (10.7%), the Generic Quality of Life Inventory-74 (GHRQOLI-74) in two studies (7.1%), the World Health Organization Quality of Life—BREF (WHOHRQOL-BREF) in two studies (7.1%), the World Health Organization Quality of Life (WHOHRQOL-100) in one study (3.6%) and the 36-Item Short Form Survey (SF-36) in one study (3.6%).

**Table 1:** Characteristics of included studies.

Author, Year	Country	Economic level	Study design	Sample size (Respondent rate)	Participant	Questionnaire
Hwang, 2004 (25)	Thailand	Upper-middle-income	Cross-sectional study	233 (80%)	BC patients	WHOHRQOL
Lu, 2007 (26)	China	Upper-middle-income	Prospective cohort study	2236 (86%)	Newly diagnosed BC patients	GHRQOLI-74
Wong, 2007 (27)	China	Upper-middle-income	Longitudinal study	249 (88%)	BC patients	FACT-G
Lu, 2009 (28)	China	Upper-middle-income	Longitudinal study	1847 (82.9%)	Newly diagnosed BC patients	GHRQOLI-74
Wong, 2009 (29)	China	Upper-middle-income	Longitudinal study	251 (49%)	BC patients	FACT-G
Matalqah, 2011 (30)	Malaysia	Upper-middle-income	Cross-sectional study	150 (100%)	BC patients	EQ-5D
Yusuf, 2013 (31)	Malaysia	Upper-middle-income	Cross-sectional study	73 (96.1%)	Newly diagnosed BC patients	EORTC QLQ-C30 EORTC QLQ-BR23
Iskandarsyah, 2013 (32)	Indonesia	Upper-middle-income	Cross-sectional study	70 (91%)	BC patients	WHOHRQOL-BREF
Hong-li, 2014 (33)	China	Upper-middle-income	Cross-sectional study	154 (100%)	BC patients	FACT-G FACT-B
Zou, 2014 (34)	China	Upper-middle-income	Cross-sectional study	156 (86.7%)	BC patients	FACT-B
Li, 2015 (35)	China	Upper-middle-income	Cross-sectional study	621 (90.8%)	BC patients	FACT-B
Ng, 2015 (36)	China	Upper-middle-income	Prospective cohort study	221 (100%)	BC patients	EORTC QLQ-C30 EORTC QLQ-BR23
Ganesh, 2016 (37)	Malaysia	Upper-middle-income	Cross-sectional study	223 (92.1%)	BC patients	EORTC QLQ-C30 EORTC QLQ-BR23
Zhang, 2017 (38)	China	Upper-middle-income	Cross-sectional study	98 (81.67%)	BC patients	FACT-B
Tang, 2017 (39)	China	Upper-middle-income	Cross-sectional study	254 (94.8%)	BC patients	EORTC QLQ-C30
Xia, 2017 (40)	China	Upper-middle-income	Cross-sectional study	10794 (100%)	BC patients	EORTC QLQ-C30 EORTC QLQ-BR23
Ahadzadeh, 2018 (17)	Malaysia	Upper-middle-income	Cross-sectional study	135 (100%)	BC patients	FACT-B
Chen, 2018 (15)	China	Upper-middle-income	Cross-sectional study	608 (97.9%)	BC patients	EORTC QLQ-C30 EORTC QLQ-BR23
Lei, 2018 (16)	China	Upper-middle-income	Prospective cohort study	1300 (88.9%)	BC patients	EORTC QLQ-C30
Wang, 2018 (41)	China	Upper-middle-income	Cross-sectional study	2626 (84%)	BC patients	EQ-5D-3l
An, 2019 (18)	China	Upper-middle-income	Cross-sectional study	382 (91%)	BC patients	FACT-B
Shin, 2020 (19)	Malaysia	Upper-middle-income	Cross-sectional study	179 (79%)	BC patients	EORTC QLQ-C30
Yang, 2020 (20)	China	Upper-middle-income	Cross-sectional study	446 (100%)	BC patients	FACT-B EQ-5D-5l EQ-VAS
Pandey, 2005 (42)	India	Lower-middle-income	Cross-sectional study	504 (100%)	BC patients	FACT-B
Jayasekara, 2008 (43)	Sri Lanka	Lower-middle-income	Cross-sectional study	356 (100%)	Newly diagnosed BC patients	EORTC QLQ-BR23
Kaur N, 2014 (44)	India	Lower-middle-income	Cross-sectional study	154 (100%)	BC patients	FACT-G FACT-B
Gangane, 2017 (45)	India	Lower-middle-income	Cross-sectional study	208 (54.17%)	BC patients	WHOHRQOL-BREF
Huong, 2019 (46)	Vietnam	Lower-middle-income	Cross-sectional study	200 (100%)	BC patients	SF-36

### 3.3. Risk of bias assessment

Out of 22 cross-sectional studies, 12 studies (54.5%) (15, 17–19, 30, 35, 37, 39, 41, 43–45) had a low risk of bias (7–9 points), and ten studies (45.5%) (20, 25, 31–34, 38, 40, 42, 46) had a fair risk of bias (4–6 points). All four prospective cohort studies (16, 26, 28, 36) had low risk of bias (7–9 points) and two longitudinal studies (27, 29) had low risk of bias (7–9 points). Details of the quality assessment results were shown in Supplementary Material S2.

### 3.4. Quality of life score in breast cancer patients

Among the 28 studies, eight studies conducted in upper-middle-income countries and used EORTC QLQ-C30 (15, 16, 19, 31, 36, 37, 39, 40). Global Health Status (GHS) score ranged from 53.8 ± 14.7 (15) to 72.48 ± 15.68 (36). Ng. and colleagues reported that the GHS of breast cancer patients increased after every 6 months follow-up (36). Breast cancer patients with higher adherence to treatment also achieved higher HRQoL scores in Lei’s study (16) (Table 2).

**Table 2:** Quality of life scores of EORTC QLQ-C30.

Author	Year	Quality of life score (Mean ± SD)					
		GHS	PF	RF	EF	CF	SF
Yusuf (31)	2013	*Malay* 60.4 (22.15) *Chinese* 65.0 (26.58)	*Malay* 76.32 (25.48) *Chinese* 84.0 (30.11)	*Malay* 67.24 (39.85) *Chinese* 80.0 (35.19)	*Malay* 65.8 (26.8) *Chinese* 76.11 (17.78)	*Malay* 84.77 (19.06) *Chinese* 91.11 (1.,39)	*Malay* 75.0 (31.1) *Chinese* 81.11 (26.63)
Ng (36)	2015	*Baseline* 69.83 (17.23) *6-month follow-up* 70.56 (16.61) *1-year follow-up* 72.48 (15.68)	*Baseline* 91.58 (13.87) *6-month follow-up* 87.77 (15.02) *1-year follow-up* 72.48 (15.68)	*Baseline* 93.21 (14.18) *6-month follow-up* 90.11 (19.03) *1-year follow-up* 91.59 (15.65)	*Baseline* 78.17 (20.44) *6-month follow-up* 83.7 (21.53) *1-year follow-up* 86.89 (16.99)	*Baseline* 89.44 (15.7) *6-month follow-up* 86.26 (18.47) *1-year follow-up* 89.44 (15.7)	*Baseline* 91.18 (18.64) *6-month follow-up* 92.49 (15.72) *1-year follow-up* 94.47 (12.98)
Ganesh (37)	2016	65.7 (21.4)	81.7 (17.6)	82.3 (25.2)	78.5 (19.9)	84.1 (18.0)	81.6 (21.8)
Tang (39)	2017	56.32 (25.42)	68.92 (31.44)	60.05 (31.71)	73.37 (22.02)	74.66 (21.71)	72.82 (21.62)
Xia (40)	2017	66.09 (23.04)	84.47 (13.59)	90.31 (17.06)	84.17 (17.02)	80.51 (17.75)	81.47 (21.79)
Chen (15)	2018	53.8 (14.7)	75.5 (17.2)	77.4 (25.5)	74.2 (19.7)	76.9 (19.5)	69.9 (24.6)
Lei (16)	2018	*WCRF/AICR Adherence Score: ≤3* 64.25 (0.92)* 3.5–4 65.58 (0.84)* >4 67.34 (0.8)*	*WCRF/AICR Adherence Score: ≤3* 89.0 (0.59)* 3.5–4 90.3 (0.54)* >4 92.18 (0.52)*	*WCRF/AICR Adherence Score: ≤3* 93.58 (0.75)* 3.5–4 93.79 (0.69)* >4 95.78 (0.66)*	*WCRF/AICR Adherence Score: ≤3* 84.11 (0.97)* 3.5–4 85.7 (0.89)* >4 85.53 (0.85)*	*WCRF/AICR Adherence Score: ≤3* 81.16 (0.99)* 3.5–4 79.53 (0.9)* >4 80.02 (0.86)*	*WCRF/AICR Adherence Score: ≤3* 93.58 (0.81)* 3.5–4 93.73 (0.74)* >4 92.92 (0.7)*
Shin (19)	2020	69.12	78.28	75.36	72.65	75.56	78.43

GHS, global health status; PF, physical functioning; CF, cognitive functioning; RF, role functioning; EF, emotional functioning; SF, social functioning.

* LS Mean ± SE.

Among six studies using EORTC QLQ-BR23 questionnaire (15, 31, 36, 37, 40, 43), four assessed HRQoL together with EORTC QLQ-C30 questionnaire (31, 36, 37, 40). Five out of six studies were conducted in upper-middle-income and Jayasekara’s study (43) was conducted in Sri Lanka—a lower-middle-income country. The body image score ranged from 64.9 ± 25 (15) to 94.85 ± 13.2 (36), the sexual functioning score ranged from 8.19 ± 16.28 (43) to 92.72 ± 14.38 (40), the sexual enjoyment score ranged from 40.9 ± 28.8 (37) to 91.85 ± 17.29 (40), and the future perspective score ranged from 44.25 ± 30.2 (31) to 66.29 ± 26.94 (43) (Supplementary Material S3).

Four studies (27, 29, 33, 44) used the FACT-G questionnaire to estimate HRQoL in breast cancer patients. Among them, three studies (27, 29, 33) were conducted in upper-middle-income countries and the remaining study (44) in lower-middle-income countries. The overall HRQoL score ranged from 60.78 ± 13.27 (33) to 82.23 ± 12.55 (27). FACT-B was used in nine studies (17, 18, 20, 33–35, 38, 42, 44), including seven studies in upper-middle-income countries (17, 18, 20, 33–35, 38) and two studies in lower-middle-income country (42, 44). The overall HRQoL score ranged from 70.29 ± 13.33 (20) to 108.48 ± 19.82 (17) (Supplementary Material S3).

### 3.5. Factors associated with HRQoL score in breast cancer patients

Factors associated with HRQoL in breast cancer patients reported in the selected studies are presented in Supplementary Material S4.

#### 3.5.1. Age

There were seven studies (15, 28, 33, 37, 44–46) reported the effect of age on HRQoL in breast cancer patients. Five studies (33, 37, 44–46) reported that older patients had higher HRQoL whereas the other two studies (15, 28) showed the opposite results. Ganesh’s study showed post- menopausal patients had better quality of life than premenopausal patients (37). Hence, the impact of age on HRQoL in breast cancer patients was inconsistent across these studies.

#### 3.5.2. Educational level

Nine studies (15, 20, 28, 32, 33, 37, 41, 44, 45) examined the association of educational level with HRQoL in breast cancer patients. The positive effect of educational attainment on HRQoL was found in eight studies (15, 20, 28, 32, 33, 41, 44, 45). In contrast, Ganesh’s study (37) reported that patients who gained primary education or less had better HRQoL than those who achieved a higher education level. Besides the patient’s education level, Pandey reported that the education level of the patient’s husband also had an influence on the patient’s HRQoL; however, the author did not mention the type of effect (42).

#### 3.5.3. Occupation

As reported in nine studies (15, 29, 32, 33, 37, 39, 41, 44, 45), the majority of studies (six studies) showed that employed patients had better HRQoL than unemployed counterparts. However, Tang (39) reported that unemployed or laid-off patients had better HRQoL than employed patients. Public employers or retirees had better HRQoL (41) as compared to manual patients (45).

#### 3.5.4. Income

Besides occupation, income also had a significant impact on the HRQoL of breast cancer patients which were reported in seven studies (20, 26, 38, 40, 41, 45, 46). All studies showed consistent findings that income had a positive effect on the HRQoL of breast cancer patients. The higher income, the better the HRQoL.

#### 3.5.5. Marital status

Seven studies (20, 29, 35, 37, 44–46) explored the association of marital status and HRQoL. Five studies showed that married women had better HRQoL than unmarried or divorced counterparts (20, 29, 35, 44, 45) while the remaining studies, Ganesh (37) and Huong (46), reported the contrast findings, which married breast cancer patients had lower HRQoL than single breast cancer patients.

#### 3.5.6. Tumor stage and treatment therapy

Patients diagnosed with breast cancer one year or more before the time of the survey had better HRQoL than those diagnosed within one year (38). The effect of tumor stage on HRQoL in breast cancer patients was reported in ten studies (15, 19, 20, 26, 28, 33, 38, 41, 42, 44). All but one study (15, 20, 26, 28, 38, 41, 42, 44 33), show that patients with advanced stage had lower HRQoL. Shin’s study (19) found that patients with advanced stage of breast cancer (stage III-IV) had better HRQoL than those with early-stages (stage I–II). Patients with metastatic or recurrent tumors had lower HRQoL than patients without metastasis (20, 28).

In terms of treatment therapy, Zhang (38) and Ganesh (37) reported that patients who underwent breast-conserving surgery had better HRQoL. Treatment with chemotherapy lowered patients’ HRQoL (19, 26, 33, 35). Patients undergoing chemotherapy had lower HRQoL than patients who had completed or did not receive chemotherapy (26). Wang (41) reported that patients receiving chemotherapy or postoperative chemotherapy had better HRQoL than patients treated with surgery alone. Patients who completed or did not begin breast cancer treatment had better HRQoL than those in the treatment process (26, 37, 41). Huong and colleagues (46) found that the treatment duration longer than 6 months had a negative effect on the HRQoL of cancer patients. Symptoms due to the disease (39) or the systemic therapy side effects (40) also had a negative effect on the HRQoL. Additionally, Lu et al. (26, 28) reported that comorbidities had a negative effect on patients’ HRQoL.

#### 3.5.7. Other factors

Patients who had anxiety, depression (18, 36), psychological distress (27), and uncertainty (17) had lower HRQoL scores, while optimistic patients (29, 34), had disease awareness (34), experienced active emotional (17) had a better HRQoL. A positive effect on HRQoL in patients with breast cancer was also from social support (34, 36, 38). Religion showed inconsistency in the association with HRQoL (29, 37, 45). A healthy lifestyle (16), do not stay up late (46), or normal BMI (18.5–22.9 kg/m^2^) (16, 26) had a positive effect on HRQoL in breast cancer patients.

## 4. Discussion

To synthesize the HRQoL and factors associated with HRQoL in breast cancer patients across LMICs in Asia, a total of 28 studies met the criteria and were included in this systematic review. Most of these studies were conducted in upper-middle-income countries. The questionnaires used to assess HRQoL were mainly EORTC QLQ-C30, EORTC QLQ-BR23 and FACT-B. The GHS score according to the EORTC QLQ-C30 questionnaire varied from 53.8 ± 14.7 (15) to 72.48 ± 15.68 (36) and the overall HRQoL score of the FACT-B questionnaire fluctuated from 70.29 ± 13.33 (20) to 108.48 ± 19.82 (17). This study indicated a consistent effect of the patient’s income on the HRQoL, the higher income, the better HRQoL. Whereas the other factors, including age, educational level, occupation, marital status, tumor stage, and treatment therapy, had an inconsistent impact on HRQoL in breast cancer patients.

Overall, the HRQoL of breast cancer patients in Asia was lower than the general population. This finding was consistent with the prior systematic review conducted in Asia (14). In addition, breast cancer patients in LMICs had lower HRQoL than those in high-income countries in Asia. Compared with the studies that used the EORTC QLQ-C30 questionnaire in this systematic review, the GHS scores in most of the studies were lower than that in studies performed in Japan (69.3 ± 18.9) (47). There are several reasons for this disparity. First, it can come from differences in patients’ characteristics, study designs, or study setting in original studies. Second, the difference in socio-economic status and health care in LMICs and high-income countries could be the reason for this disparity. In LMICs in Asia such as China and India, the majority of breast cancer patients live in rural areas which prevent them from accessing appropriate treatment (48). Insufficient resources for cancer screening which lead to the majority of cancer patients being diagnosed at an advanced stage, then negatively affects HRQoL of patients in LMICs compared to high-income countries (48). Additionally, a high cost of breast cancer treatment with low household income also worsen patients’ HRQoL in these countries (49).

The effect of age on HRQoL in breast cancer patients varied among included studies. The vast majority of studies showed that older patients had higher QoL (33, 37, 44–46), which, Ganesh (37) made clearer when pointed out that post-menopausal breast cancer patients had better QoL than premenopausal one. With the same finding, Yeo et al. also showed that patients who experienced worse menopausal symptoms had low QoL (50). However, data on the relationship between menopausal status and HRQoL in breast cancer patients in LMICs in Asia is still lacking; more in-depth research on this relationship is needed to reach a clear conclusion.

This systematic review found that patients with a high income had better HRQoL than those with low income (20, 26, 38, 40, 41, 45, 46). This result was consistent with the previous study in Shang Hai, China (51). Apparently, once patients can pay for their treatment without financial hardship, they are more likely to adhere to the treatment and get better outcomes as consequence. In contrast, if the patients are unable to afford treatment, they will suffer worse outcomes. Health insurance is one of the most effective financial support solutions for breast cancer patients. However, there is an actual issue: the health insurance system in LMICs in Asia does not cover all breast cancer treatment fees (surgeries and medicines). In Vietnam, while other healthcare services and medicines for breast cancer are paid by Vietnam Social Health Insurance, Trastuzumab are only covered 40%–60% (depends on type of breast cancer); Pertuzumab and breast reconstruction surgery are not in the cover list. The results are breast cancer patients had to pay a large amount of out-of-pocket costs for the treatment which caused catastrophic costs to patients in Vietnam (52). In China, the health insurance coverage rate for drugs and services for breast cancer is still inadequate, resulting in patients incurring large catastrophic health expenditures (53). To improve health outcomes and health-quality of life in breast cancer patients, the government, policymakers, and other relevant parties in LMICs in Asia should consider including anti-cancer medications and surgeries in health insurance coverage.

Additionally, having a stable occupation also had a positive effect on HRQoL. However, Tang reported that breast cancer patients who were laid off or unemployed had better HRQoL than those who were employed (39). Given the secondary data derived in this review, this inconsistent effect of occupation on HRQoL in breast cancer patients in LMICs in Asia could not be explored.

In terms of educational level, the majority of studies (eight out of nine studies) reported that the HRQoL was better in highly educated patients than in less educated or uneducated patients (15, 20, 28, 32, 33, 41, 44, 45). Not only affected by their own education, the HRQoL of breast cancer patients were also influenced by their husband’s education (42). Education had a positive effect on breast cancer diagnosis and screening. When women were well educated and had a better knowledge of breast cancer and its treatment, they were more aware of the vital role that early breast cancer screening, diagnosing and getting treatment (54).

There was a conflict result in the effect of marital status on HRQoL in breast cancer patients in LMICs. When most studies reported that women who were married or in a relationship had higher HRQoL than those who were divorced or single (20, 29, 35, 44, 45), Huong’s study (46) and Ganesh’s study (37) reported that marriage had a negative effect on HRQoL. A study on breast cancer survivors after 5 years of diagnosis found that married patients had higher levels of optimism than unmarried women (55) and optimism had a positive effect on the HRQoL of breast cancer patients (29, 34). Another study reported that married breast cancer patients had lower mortality than single or divorced patients (56).

Patients with advanced stage of breast cancer reported lower HRQoL than those with the early stage when reported in eight studies (15, 20, 26, 28, 33, 38, 41, 44). However, Shin et al. (19) reported the opposite result. The effect of early stage on HRQoL in breast cancer was also reported in a previous systematic review (14). This finding may imply that if women’s health awareness in terms of the role of early detection of breast cancer (57), they may get better outcomes and better HRQoL. In addition, LMICs in Asia need to break the barriers to screening for breast cancer. One of the most challenging for early breast cancer detection is the cost of screening (4). A systematic review has found that mammography is a cost-effective breast cancer screening method in LMICs in Asia, especially in upper-middle-income countries with the target population of 50–59 years (58).

Vietnam and other LIMCs in Asia have implemented Universal Health Coverage (UHC) and targeted to achieve UHC in the year 2030 (59, 60). A study found that sufficient use of fruit and vegetables is the most important prevention indicator in reaching the Universal Health Coverage (UHC) goal in non-communicable disease (NCD) management in Vietnam (59). Additionally, there are several obstacles in reaching the goal of coverage for reproductive, maternal, newborn, and child health (RMNCH) services (61). Preventing diseases and conducting early screenings are essential in reducing the impact of late-stage NCD diagnoses and achieving the UHC goal, particularly in LMICs. To reach this target, the government should consider covering the cost of health services, increasing public awareness, and issuing a call to action.

The longer the patient was treated, the lower the HRQoL. The studies included in this systematic review showed that patients undergoing treatment had lower HRQoL than patients who had not undergone treatment or completed cycles of treatment (26, 37, 41). Bhandari et al. also reached a similar conclusion when reported that patients on treatment had poorer HRQoL (62). Long duration of treatment also negatively affects the HRQoL of patients with breast cancer (46). In addition, breast cancer patients with comorbidities had poorer HRQoL than those without comorbidities (28). This result was also proved through the Fu’s study (63). This could be due to the influence of symptoms and treatment of comorbidities on the physical and mental well-being of cancer patients.

According to Lu’s study, breast cancer patients who were receiving chemotherapy had a lower quality of life than those who had completed chemotherapy (26). This can be explained by the fact that breast cancer patients had to deal with several drug adverse events that could reduce their quality of life such as edema, myalgia, nail problem, febrile neutropenia, upper respiratory tract infection, decreased appetite, and rash (64).

The effects of educational level, occupation, marital status, tumor stage, and treatment therapy on HRQoL in breast cancer patients in LMICs in Asia were inconsistent across the studies. Given that fully based on original studies for data extraction, we could not identify the reason for this discrepancy. There are several reasons that can be scrutinized for this. First, the characteristics of patients in these studies were largely heterogeneous. Second, the HRQoL assessment tools were different in these studies which may affect the HRQoL score and the associated factors. Third, the time of included studies ranged from 2004 (25) to 2020 (19, 20), so the assessment of the impact on HRQoL may not be the same for each factor. Moreover, the small sample size in some studies could lead to less power to detect the significance of associations.

However, none of included studies reported on the relationship between the histologic type of breast cancer and HRQoL in patients from LMICs in Asia. The risk of mortality varied throughout histologic type of breast cancer, in which, invasive ductal carcinoma (IDC) had the highest risk compared to others (65). In contrast, another study found that higher risk of mortality was found in invasive lobular carcinoma (ILC) patients who had HR (-), AJCC stage III, N2/N3 stage, or in those who received radiotherapy (66). In order to gain a deeper understanding of the impact of histologic type on HRQoL in breast cancer patients, it is suggested that further research be conducted in this topic, with an emphasis on LMICs.

This systematic review also had some limitations. Firstly, the findings and the quality of review completely relied on secondary data in the included studies. To ensure the validity of findings, however, we also conducted an assessment of bias for the studies. Secondly, the HRQoL was summarized and synthesized for breast cancer patients in all stages, so the results should be interpreted with caution for breast cancer patients in a specific stage or specific treatment.

## 5. Conclusion

Breast cancer patients in LMICs in Asia experienced lower HRQoL than the general population. Patients with high incomes had better HRQoL compared to those who had lower incomes. HRQoL of these patients was also affected by several sociodemographic factors which should be studied more in future research.

## Data Availability

The original contributions presented in the study are included in the article/**Supplementary Material**, further inquiries can be directed to the corresponding author.
