# Supplementary material for: Health-related quality of life in breast cancer patients in low-and-middle-income countries in Asia: a systematic review
Source: Front Glob Womens Health. 2023 Jun 14;4:1180383. doi: 10.3389/fgwh.2023.1180383 (PMC10304018; doi:10.3389/fgwh.2023.1180383)
Supplement: Supplementary file 1 [file Table1.docx]

Supplementary Material

Health-related quality of life in breast cancer patients in

low-and-middle-income countries in Asia: A systematic review

Nhi T.N. Ngo^1^, Ha Thi Nguyen^1^, Phuong Thi Lan Nguyen^1^, Truc Thuy Thanh Vo^1^, Toi Lam Phung^2^, Anh Gia Pham^3^, Thanh Van Vo^4,5^ , Mai Thi Ngoc Dang^6^, Tien Nguyen Le Bao^5^, Khanh N.C. Duong^1,7*^

^1^School of Medicine, Vietnam National University, Ho Chi Minh City 70000, Vietnam

^2^Health Strategy and Policy Institute, Ministry of Health, Ha Noi 10000, Vietnam

^3^Oncology Department, Viet Duc Hospital, Hanoi 10000, Vietnam

^4^Department of Surgery, Hanoi Medical University, Hanoi 10000, Vietnam

^5^Institute of Orthopedics and Trauma Surgery, Viet Duc Hospital, Hanoi 10000, Vietnam

^6^Center of clinical pharmacology, Hanoi Medical University, Hanoi 10000, Vietnam

^7^Department of Pharmacotherapy, College of Pharmacy, University of Utah, Utah, United States

*** Correspondence:**Khanh N.C. Duong
[dnckhanh@medvnu.edu.vn](mailto:dnckhanh@medvnu.edu.vn)

# Table S1: Search terms and search strategy in databases

## Search strategy in PubMed (Date of search: 19-Nov-2020)

| **No.** | **Search strategies** |
| --- | --- |
|  | breast neoplasm[MeSH Terms] |
|  | "breast cancer*" |
|  | "breast neoplasm*" |
|  | "breast tumor*" |
|  | "breast carcinoma*" |
|  | #1 OR #2 OR #3 OR #4 OR #5 |
|  | "quality of life" |
|  | "QoL" |
|  | "HRQoL" |
|  | "patient-report outcome*" |
|  | #7 OR #8 OR #9 OR #10 |
|  | Asia |
|  | Asia* |
|  | “southeast asia” |
|  | Vietnam* |
|  | timor* |
|  | thai* |
|  | philippin* |
|  | Nepal* |
|  | Mongoli* |
|  | Malay* |
|  | (burmese) OR (myanmar*) |
|  | Maldives |
|  | ((laos) OR (lao)) OR ("Lao People's Democratic Republic") |
|  | cambodia* |
|  | (india) OR (india*) |
|  | indonesia* |
|  | (china) OR (chine*) |
|  | bhutan* |
|  | bangladesh* |
|  | ("democratic people's republic of korea") OR ("north korea") |
|  | "sri lanka*" |
|  | #12 OR #13 OR #14 OR #15 OR #16 OR #17 OR #18 OR #19 OR #20 OR #21 OR #22 OR #23 OR #24 OR #25 OR #26 OR #27 OR #28 OR #29 OR #30 OR #31 OR #32 |
|  | #6 AND #11 AND #33 |

## Search strategy in Cochrane (Date of search: 18-Nov-2020)

| **No.** | **Search strategies** |
| --- | --- |
|  | MeSH descriptor: [Breast Neoplasms] explode all trees |
|  | ("breast cancer") |
|  | ("breast neoplasm") |
|  | ("breast tumor") |
|  | ("breast carcinoma") |
|  | #1 OR #2 OR #3 OR #4 OR #5 |
|  | ("quality of life") |
|  | ("QoL") |
|  | ("HRQoL") |
|  | ("patient reported outcome") |
|  | #7 OR #8 OR #9 OR #10 |
|  | MeSH descriptor: [Asia] explode all trees |
|  | ("Asia") |
|  | ("Southeast Asian") |
|  | ("Southeastern Asia") |
|  | (vietnam*) |
|  | (timor*) |
|  | (thai*) |
|  | ("Sri Lanka") OR ("sri lanka*") |
|  | ("Philippine") OR (philippine*) |
|  | (nepal*) |
|  | (mongoli*) |
|  | (malay*) |
|  | ("Myanmar") OR ("Burmese") |
|  | (maldives) |
|  | (lao*) |
|  | ("Cambodia") OR (cambodia*) |
|  | ("India") OR (india*) |
|  | (indonesia*) |
|  | ("China") OR ("Chinese") |
|  | (bhutan*) |
|  | (bangladesh*) |
|  | ("democratic People's Republic of korea") OR ("north korea") |
|  | #12 OR #13 OR #14 OR #15 #16 #17 OR #18 #19 OR #20 #21 OR #22 OR #23 OR #24 #25 OR #26 OR #27 #28 #29 #30 OR #31 OR #32 #33 |
|  | #6 AND #11 AND #34 |

## Search strategy in Scopus (Date of search: 19-Nov-2020)

| **No.** | **Search strategies** |
| --- | --- |
| #1 | TITLE-ABS-KEY ("breast cancer*") |
| #2 | TITLE-ABS-KEY ("breast neoplasm*") |
| #3 | TITLE-ABS-KEY ("breast tumor*") |
| #4 | TITLE-ABS-KEY ("breast carcinoma*") |
| #5 | #1 OR #2 OR #3 OR #4 |
| #6 | TITLE-ABS-KEY ("quality of life") |
| #7 | TITLE-ABS-KEY (QOL) |
| #8 | TITLE-ABS-KEY (HRQOL) |
| #9 | TITLE-ABS-KEY ("patient-report outcome*") |
| #10 | #6 OR #7 OR #8 OR #9 |
| #11 | TITLE-ABS-KEY (Asia*) |
| #12 | TITLE-ABS-KEY (Vietnam*) |
| #13 | TITLE-ABS-KEY (timor*) |
| #14 | TITLE-ABS-KEY (thai*) |
| #15 | TITLE-ABS-KEY (philippin*) |
| #16 | TITLE-ABS-KEY (Nepal*) |
| #17 | TITLE-ABS-KEY (Mongoli*) |
| #18 | TITLE-ABS-KEY (Malay*) |
| #19 | TITLE-ABS-KEY (Burmese) |
| #20 | TITLE-ABS-KEY (myanmar*) |
| #21 | TITLE-ABS-KEY (Maldives) |
| #22 | TITLE-ABS-KEY (lao*) |
| #23 | TITLE-ABS-KEY (cambodia*) |
| #24 | TITLE-ABS-KEY (india*) |
| #25 | TITLE-ABS-KEY (indonesia*) |
| #26 | TITLE-ABS-KEY (china) |
| #27 | TITLE-ABS-KEY (chine*) |
| #28 | TITLE-ABS-KEY (bhutan*) |
| #29 | TITLE-ABS-KEY (bangladesh*) |
| #30 | TITLE-ABS-KEY ("democratic people's republic of korea") |
| #31 | TITLE-ABS-KEY ("north korea") |
| #32 | TITLE-ABS-KEY (“sri lanka*”) |
| #33 | #11#12 OR #13 OR #14 OR #15 OR #16 OR #17 OR #18 OR #19 OR #20 OR #21 OR #22 OR #23 OR #24 OR #25 OR #26 OR #27 OR #28 OR #29 OR #30 OR #31 OR #32 OR #33 |
| #34 | #10 AND #5 AND #33 |

# Table S2: The Newcastle-Ottawa Scale assessment quality of observational study

| **Author** | **Year** | **Selection** | **Comparability** | **Outcome** | **Total score** | **Appraisal score** |
| --- | --- | --- | --- | --- | --- | --- |
| **Cross-sectional study** | | | | | | |
| Hwang | 2004 | 4 | 0 | 2 | **6** | Fair |
| Matalqah | 2011 | 3 | 2 | 2 | **7** | Low |
| Yusuf | 2013 | 3 | 0 | 2 | **5** | Fair |
| Iskandarsyah | 2013 | 3 | 1 | 2 | **6** | Fair |
| Hong-li | 2014 | 3 | 1 | 2 | **6** | Fair |
| Zou | 2014 | 3 | 1 | 2 | **6** | Fair |
| Li | 2015 | 4 | 2 | 2 | **8** | Low |
| Ganesh | 2016 | 4 | 1 | 2 | **7** | Low |
| Zhang | 2017 | 3 | 1 | 2 | **6** | Fair |
| Tang | 2017 | 5 | 1 | 2 | **8** | Low |
| Xia | 2017 | 3 | 1 | 2 | **6** | Fair |
| Ahadzadeh | 2018 | 4 | 1 | 2 | **7** | Low |
| Chen | 2018 | 5 | 1 | 2 | **8** | Low |
| Wang | 2018 | 5 | 2 | 2 | **9** | Low |
| An | 2019 | 5 | 1 | 2 | **8** | Low |
| Shin | 2020 | 4 | 1 | 2 | **7** | Low |
| Yang | 2020 | 3 | 1 | 2 | **6** | Fair |
| Pandey | 2005 | 3 | 0 | 1 | **4** | Fair |
| Jayasekara | 2008 | 4 | 1 | 2 | **7** | Low |
| Kaur | 2014 | 5 | 1 | 2 | **8** | Low |
| Gangane | 2017 | 5 | 1 | 2 | **8** | Low |
| Huong | 2019 | 4 | 0 | 2 | **6** | Fair |
| **Longitudinal study** | | | | | | |
| Wong | 2007 | 4 | 2 | 3 | **9** | Low |
| Wong | 2009 | 4 | 0 | 3 | **7** | Low |
| **Prospective cohort study** | | | | | | |
| Lu | 2007 | 4 | 1 | 2 | **7** | Low |
| Lu | 2009 | 4 | 0 | 3 | **7** | Low |
| Ng | 2015 | 4 | 1 | 3 | **8** | Low |
| Lei | 2018 | 4 | 1 | 3 | **8** | Low |

# Table S3: Quality of life score of breast cancer patients

## Quality of life score of EORTC QLQ-BR23

| **Author** | **Year** | **Quality of life score (Mean ± SD)** | | | |
| --- | --- | --- | --- | --- | --- |
|  |  | **Body image** | **Sexual functioning** | **Sexual enjoyment** | **Future perspective** |
| Yusuf | 2013 | ***Malay***  75.57 (26.53)  ***Chinese***  81.11 (29.46) | ***Malay***  77.3 (25.69)  ***Chinese***  77.78 (24.12) | ***Malay***  50.0 (21.28)  ***Chinese***  58.33 (29.55) | ***Malay***  44.25 (30.2)  ***Chinese***  57.78 (29.46) |
| Ng | 2015 | ***Baseline***  92.61 (16.27)  ***6-month follow-up***  91.39 (17.39)  ***1-year follow-up***  94.85 (13.21) |  |  |  |
| Ganesh | 2016 | 80.0 (24.6) | 14.3 (23.1) | 40.9 (28.8) | 59.8 (32.6) |
| Xia | 2017 | 67.63 (24.89) | 92.72 (14.38) | 91.85 (17.29) | 62.0 (31.72) |
| Chen | 2018 | 64.9 (25.0) | 89.0 (15.9) | 88.3 (19.6) | 51.5 (31.4) |
| Jayasekara | 2008 | 80.66 (20.4) | 8.19 (16.28) | 41.22 (17.09) | 66.29 (26.94) |

## Quality of life score of FACT-G, FACT-B

| **FACT-G; FACT-B** | | |
| --- | --- | --- |
| **Author** | **Year** | **Total Quality of life score (Mean ± SD)** |
| Wong | 2007 | **FACT-G**  ***Baseline:*** 77.67 (14.91)  ***3-month follow-up:*** 79.58 (14.22)  ***6-month follow-up:*** 82.23 (12.55) |
| Wong | 2009 | **FACT-G**  ***Baseline:*** 77.51 (15.09)  ***3-month follow-up:*** 79.58 (14.22)  ***6-month follow-up:*** 81.93 (12.83) |
| Hong-li | 2014 | **FACT-G**  ***1 year follow-up:*** 60.78 (13.27)  ***2 years folow-up:*** 67.22 (18.96)  ***5 years follow-up:*** 66.87 (10.76)  **FACT-B**  ***1 year follow-up:*** 81.06 (14.6)  ***2 years folow-up:*** 85.75 (20.15)  ***5 years follow-up:*** 88.83 (12.8) |
| Kaur | 2014 | **FACT-G**  ***1 year follow-up:*** 60.78 (13.27)  ***2 years folow-up:*** 67.22 (18.96)  ***5 years follow-up:*** 66.87 (10.76)  **FACT-B**  ***1 year follow-up:*** 81.06 (14.6)  ***2 years folow-up:*** 85.75 (20.15)  ***5 years follow-up:*** 88.83 (12.8) |
| Zou | 2014 | **FACT-B:** 95.48 (18.19) |
| Li | 2015 | **FACT-B:** 84.8 (18.98) |
| Zhang | 2017 | **FACT-B:** 80.74 (19.49) |
| Ahadzadeh | 2018 | **FACT-B:** 108.48 (19.82) |
| An | 2019 | **FACT-B:** 96.42 (17.79) |
| Yang | 2020 | **FACT-B:** 70.29 (13.33) |
| Pandey | 2005 | **FACT-B:** 90.5 (18.4) |

## Quality of life score of other questionnaires

| **EQ-5D; EQ-VAS** | | | | | |
| --- | --- | --- | --- | --- | --- |
| **Author** | **Year** | **Total Quality of life score** | | | |
| Matalqah | 2011 | **EQ-5D-3L:** 0.71 (0.25)  **EQ-VAS:** 72.7 (16.9) | | | |
| Wang | 2018 | **EQ-5D-3L:** 0.781 (0.774 – 0.788) *^a^*  **EQ-VAS:** 72.8 (72.3 – 73.3) *^a^* | | | |
| Yang | 2020 | **EQ-5D-5L:** 0.9 (0.83 – 1) *^b^*  **EQ-VAS:** 80.0 (70.0 – 90.0) *^b^* | | | |
| **GQOLI-74** | | | | | |
| **Author** | **Year** | **Total Quality of life score** | | | |
| Lu | 2007 | **Overall:** 61.8 (8.6) | | | |
| Lu | 2009 | **6-month:** 59.5 (7.9)  **36-month:** 60.6 (8.6) | | | |
| **WHOQOL-100** | | | | | |
| **Author** | **Year** | **Quality of life score** (Estimate ± SE) | | | |
|  |  | **Physical health** | **Psychological health** | **Social health** | **Environmental health** |
| Hwang | 2004 | **Stage I**  98.5 (3.3)  **Stage II**  77.0 (2.9)  **Stage III**  53.5 (3.1)  **Stage IV**  20.2 (2.5) | **Stage I**  93.4 (3.1)  **Stage II**  74.1 (2.2)  **Stage III**  50.8 (3.0)  **Stage IV**  20.9 (2.4) | **Stage I**  87.4 (3.4)  **Stage II**  75.1 (2.2)  **Stage III**  49.8 (3.0)  **Stage IV**  20.5 (2.2) | **Stage I**  91.1 (2.9)  **Stage II**  74.1 (2.2)  **Stage III**  51.3 (2.9)  **Stage IV**  20.9 (2.5) |
| **WHOQOL-BREF** | | | | | |
| **Author** | **Year** | **Total Quality of life score** | | | |
| Iskandarsyah | 2013 | **Overall:** 3.06 (0.66) | | | |
| Gangane | 2017 | **Overall:** 59.3 | | | |
| **SF-36** | | | | | |
| Huong | 2019 | **Overall:** 54.6 (52.5 – 56.7) *^a^*  **Overall:** 52.6 (43.5 – 63.1) *^b^* | | | |

*^a^*: Mean (95% CI)

*^b^*: Median (IQR)

# Table S4: Factors associated with quality of life in breast cancer patents

| **Author** | **Year** | **QoL questionnaire** | **Factors associated** | **QoL outcome** | **Type of associated** |
| --- | --- | --- | --- | --- | --- |
| Hwang | 2004 | WHOQOL-100 | _ | _ | _ |
| Lu | 2007 | GQOLI-74 | Low household income, have chronic disease | Overall, physical, psychological, social, material health | Negative |
|  |  |  | BMI (<18.5 vs 18-25); Radiotherapy (complete vs no) | Overall, physical, social health | Negative |
|  |  |  | BMI (25-30 vs 18-25) | Overall, physical, psychological health | Positive |
|  |  |  | Chemotherapy (current vs no) | Physical health | Negative |
|  |  |  | Tamoxifen (current use vs no) | Physical health | Positive |
|  |  |  | Tamoxifen (ever use vs no) | Overall, psychological, material health | Negative |
|  |  |  | Tumor stage (IIB-IV vs 0- I) | Social health | Negative |
| Wong | 2007 | FACT-G | Longer time since diagnosis | Overall, functional well-being | Positive |
|  |  |  | Positive mood | Overall, physical well-being | Positive |
|  |  |  | Higher levels of boredom | Overall, functional well-being | Negative |
| Lu | 2009 | GQOLI-74 | Age (≥ 60) | Overall health | Negative |
|  |  |  | High educational level | Psychological health | Positive |
|  |  |  | Comorbidities (Charlson score ≥3) | Social health | Negative |
|  |  |  | Radiotherapy | Overall health | Positive |
|  |  |  | Estrogen receptor/ progesterone receptor status ER/PR (mixed vs positive) | Overall, physical, psychological health | Positive |
|  |  |  | Recurrence/metastatis status (yes vs no) | Overall, physical, psychological, social health | Negative |
| Wong | 2009 | FACT-G | Marital status (nonsingle), Occupation (working), Appetite, Optimism | Overall well-being | Positive |
|  |  |  | Endorsing a religion (yes), Pain, Depressed mood | Overall well-being | Negative |
| Matalqah | 2011 | EQ-5D; EQ-VAS | _ | _ | _ |
| Yusuf | 2013 | EORTC QLQ-C30 EORTC QLQ-BR23 | _ | _ | _ |
| Iskandarsyah | 2013 | WHOQOL-BREF | Housewives/ unemployed | Physical health | Negative |
|  |  |  | Lower education | Environment health | Negative |
|  |  |  | Time since the diagnosis | Physical/ Psychological/ Environment health | Positive |
|  |  |  | Higher level of satisfaction with cancer information, illness perception | Overall QoL | Positive |
| Hong-li | 2014 | FACT-G  FACT-B | Age (< 40), Higher tumor stage | Emotional well-being | Negative |
|  |  |  | Educational level, Occupation | Social well-being | Positive |
|  |  |  | Lower tumor stage | Physical well-being | Positive |
|  |  |  | Lower tumor stage; chemotherapy and radiotherapy | Breast specific subscale | Negative |
| Zou | 2014 | FACT-B | Optimism, Social support, Disease arwaness, Lower symptom distress score, Lower appraisal of illness score (less stress), Lower give-in coping mood score | Overall well-being | Positive |
| Li | 2015 | FACT-B | Marital status (in relationship vs divorced or widowed); Place of residence (urban vs rural); Adaptive strategies (acceptance and positive reppraisal) | Overall well-being | Positive |
|  |  |  | Undergoing chemotherapy vs undergoing surgery alone; Maladaptive strategies (self-blame, rumination, catastrophizing) | Overall well-being | Negative |
| Ng | 2015 | EORTC QLQ-C30 EORTC QLQ-BR23 | Anxiety, depression | Overall QoL | Negative |
|  |  |  | Social support | Overall QoL | Positive |
| Ganesh | 2016 | EORTC QLQ-C30 EORTC QLQ-BR23 | Age (>55) | Functioning, Syptoms | Positive |
|  |  |  | Employment status (employed) | Global Health Status/ Functioning (physical functioning, emotional functioning, sexual functioning), Symptoms | Positive |
|  |  |  | Post-menopausal women | Functioning (social functioning, body image, sexual functioning, future perspective), Syptoms | Positive |
|  |  |  | Chinese | Functioning (role functioning, sexual functioning, future perspective), Syptoms | Positive |
|  |  |  | Marital status (single, divorced, or widowed compared to married) | Functioning (physical functioning, social functioning, body image, sexual functioning, future perspective), Symptoms | Positive |
|  |  |  | Educational level (primary school and lower vs secondary school and higher) | Functioning (body image), Symptoms | Positive |
|  |  |  | Follow-up (compared to in treatment) | Functioning, Symptoms | Positive |
|  |  |  | Breast conserving sutgery | Symptoms | Positive |
| Zhang | 2017 | FACT-B | High income; Social support; Time since diagnose; Early stage; Breast conserving surgery; Resilience | Overall well-being | Positive |
| Tang | 2017 | EORTC QLQ-C30 | Retired/ unemployment; Breast cancer symptoms; Emotional distress | Global Health Status | Positive |
| Xia | 2017 | EORTC QLQ-C30 EORTC QLQ-BR23 | Financial difficulties; Fatigue; Systemic therapy side effects | Global Health Status | Negative |
| Ahadzadeh | 2018 | FACT-B | Uncertainty | Overall well-being | Negative |
|  |  |  | Active emotional coping | Overall well-being | Positive |
| Chen | 2018 | EORTC QLQ-C30 EORTC QLQ-BR23 | Age (older); Higher tumor stage | Global Health Status | Negative |
|  |  |  | Residence (rural); Educational level (university degree or above); Employment status (urban employee) | Global Health Status | Positive |
| Lei | 2018 | EORTC QLQ-C30 | Healthy lifestyle | Global Health Status | Positive |
|  |  |  | Physical activity; Healthy diet; Normal BMI | Functioning | Positive |
| Wang | 2018 | EQ-5D  EQ-VAS | Region (Central); Undergraduate or over; Public sector employee or Retiree; High household income; Survey timing (post-treatment/ before treatment vs in treatment); Therapy regimen (chemotherapy, surgery and postoperative chemotherapy vs surgery) | Overall QoL | Positive |
|  |  |  | Clinical stage (stage IV vs stage I) | Overall QoL | Negative |
| An | 2019 | FACT-B | Care giver burden; Anxiety, depression | Overall well-being | Negative |
| Shin | 2020 | EORTC QLQ-C30 | Advancde stage; Mid-upper arm circumference; Handgrip strength | Global Health Status | Positive |
|  |  |  | Chemotherapy compared to radiotherapy | Role functioning | Negative |
|  |  |  | High level of serum Albumin | Physical functioning | Positive |
|  |  |  | High level of serum Hemoglobin | Socical functioning | Positive |
| Yang | 2020 | FACT-B  EQ-5D-5L  EQ-VAS | Married | Social well-being | Positive |
|  |  |  | Education above secondary school; Urban resident insurance | Overall well-being | Positive |
|  |  |  | High income | Functional well-being | Negative |
|  |  |  | State R vs P | Overall, functional, social well-being | Negative |
|  |  |  | State M vs P | Overall, emotional, socail well-being | Negative |
|  |  |  | Inpatient vs outpatient | Overall, emotional, social, physiological well-being | Negative |
|  |  |  | Radiotherapy | Overall well-being | Negative |
| Pandey | 2005 | FACT-B | The distance to treatment center | Physical well-being | Not mention |
|  |  |  | Religion | Overall, social, emotional, functional well-being | Not mention |
|  |  |  | Husband’s education | Breast specific subscale | Not mention |
|  |  |  | Metastatic tumor | Physical well-being | Not mention |
|  |  |  | Tumor stage | Overall, social, functional well-being | Not mention |
|  |  |  | Pain | Emotional well-being | Not mention |
| Jayasekara | 2008 | EORTC QLQ-BR23 | _ | _ | _ |
| Kaur | 2014 | FACT-G  FACT-B | Young age | Breast specific subscale | Negative |
|  |  |  | High education; Occupation | Socail well-being | Positive |
|  |  |  | Widowed | Emotional well-being | Negative |
|  |  |  | Early stage | Physical well-being | Positive |
|  |  |  | Weight gain after chemotherapy | Breast specific subscale | Negative |
| Gangane | 2017 | WHOQOL-BREF | Age (>50) | Environment health | Positive |
|  |  |  | Non-Hindu religion | Social health | Negative |
|  |  |  | Lower education (below secondary, illiterate) | Environment health | Negative |
|  |  |  | Housewife/ Casual worker Industry /office | Physical health | Negative |
|  |  |  | Higher income | Social, environment health | Positive |
|  |  |  | Divorced/ single | Psychological/ social health | Negative |
|  |  |  | Better self efficacy | Physical, psychological, social, environment health | Positive |
| Huong | 2019 | SF-36 | Age (>50) | Psychological health | Positive |
|  |  |  | Economic status (Poor/near poor) | Vitality | Negative |
|  |  |  | Married | General health | Positive |
|  |  |  | Stay up late habit | Overall, bodily pain, vitality, social functioning, role emotional | Negative |
|  |  |  | Stress | Overall, role physical, bodily pain, general health, vitality, social functioning, role emotional, mental health | Negative |
|  |  |  | Treatment duration > 6 months | Physical functioning | Negative |
